# Incidental Finding of Triple Ectopic Thyroid: Case Report and Review of the Literature

**DOI:** 10.22038/AOJNMB.2024.75895.1532

**Published:** 2024

**Authors:** Fatima Zeineddine, Gerard El-Hajj, Marie-Ange Hajj, Rita Chahinian

**Affiliations:** 1 Medical Imaging Department, Saint George Hospital University Medical Center, Beirut, Lebanon; 2 Saint George University of Beirut, Beirut, Lebanon; 3 University of Balamand, Beirut, Lebanon; 4 Lebanese University, Beirut, Lebanon

**Keywords:** Triple ectopic thyroid, Developmental anomaly, Technetium-99m pertechnetate, Ultrasonography

## Abstract

Ectopic thyroid tissue is a rare congenital anomaly, with the presence of three simultaneous ectopic foci being exceedingly rare. We describe a case of a totally asymptomatic 26-year-old male discovered to have triple ectopic thyroid following incidental elevated thyroid-stimulating hormone (TSH) levels. Subsequent ultrasonography of the neck showed an absent thyroid gland in its conventional location. A Technetium-99m pertechnetate (Tc-99m) thyroid scan showed three distinct foci of radiotracer uptake in the upper cervical, lingual, and sublingual regions, corresponding to triple ectopic thyroid. An extensive review of the literature was conducted to provide a broader understanding and deeper insights into this uncommon condition. This case underscores the pivotal role of Technetium-99m thyroid scanning in diagnosing triple ectopic thyroid, particularly in instances where the orthotopic thyroid gland is absent. A comprehensive understanding of this rare entity is indispensable for radiologists and clinicians, enabling accurate diagnosis and informed decision-making regarding the appropriate therapeutic strategies.

## Introduction

 Ectopic thyroid tissue represents a congenital anomaly characterized by the occurrence of thyroid tissue in sites beyond its usual cervical locations. These aberrant sites are often observed along the path of the thyroglossal duct, with the lingual thyroid being the predominant manifestation. However, instances of ectopic thyroid tissue in remote anatomical regions are infrequent. Remarkably, the conventional thyroid gland is notably absent in approximately 70% of ectopic thyroid cases (1). The emergence of triple ectopic thyroid, as a distinctive presentation of thyroid ectopia, remains a rare phenomenon, with only limited documented cases within the literature.

## Case report

**Figure 1:**
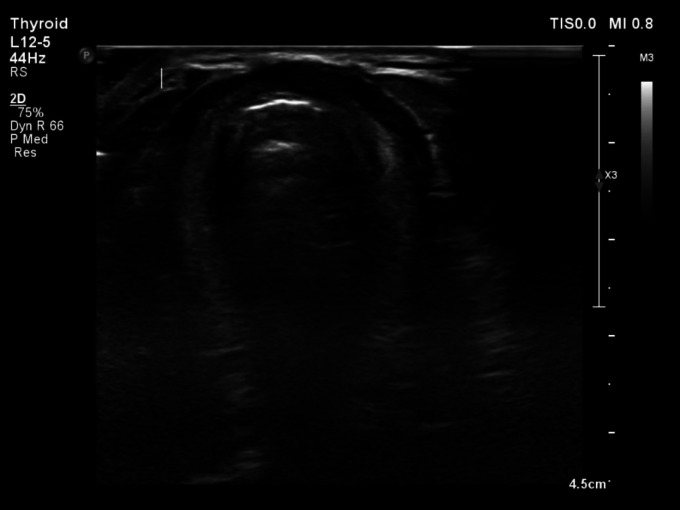
Ultrasound image of the neck demonstrating absent thyroid gland in its usual location

 A 26-year-old male presented with an incidental finding of elevated thyroid-stimulating hormone (TSH) levels, without any relevant associated symptoms. Upon local examination, the conventional cervical palpation failed to detect the thyroid gland at its typical anatomical location. Thyroid function assessments indicated subclinical hypo-thyroidism, with a TSH level measuring 11.19 mIU/L, and a free thyroxine (FT4) level of 1.03 ng/dL. Subsequent neck ultrasonography conducted using a linear array transducer (10–15 MHz), revealed the absence of thyroid gland tissue from its expected site (Figure 1).

 Subsequently, a Technetium-99m thyroid scan was conducted, revealing three distinct foci of tracer uptake. The thyroid scan was performed on a “GE Discovery NM/CT 670 Pro” using a dual-head gamma camera with a low-energy, high-resolution collimator. The matrix size for the images was set at 256×256 pixels. The SPECT/CT was conducted approximately 20 minutes post-injection to ensure optimal distribution of the Technetium-99m per-technetate (10 mCi). Each SPECT acquisition was over a 360-degree rotation with a duration of 15 seconds per step, comprising a total of 24 steps. A whole-body scan was also performed to detect any additional ectopic foci.

**Figure 2:**
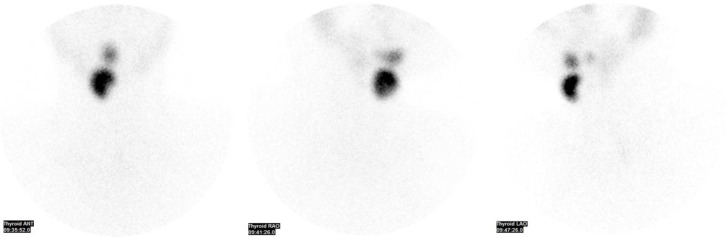
Thyroid scan with Technetium-99m revealing tracer uptake at three different levels other than the normal thyroid gland location (Anterior, and bilateral obliques images). Starting inferiorly, the first focus just below the level of the hyoid bone (infra-hyoid), the second focus was situated between the geniohyoid and mylohyoid muscles (sublingual), and the third presented as a small focus with faint uptake at the base of the tongue (lingual)

**Figure 3:**
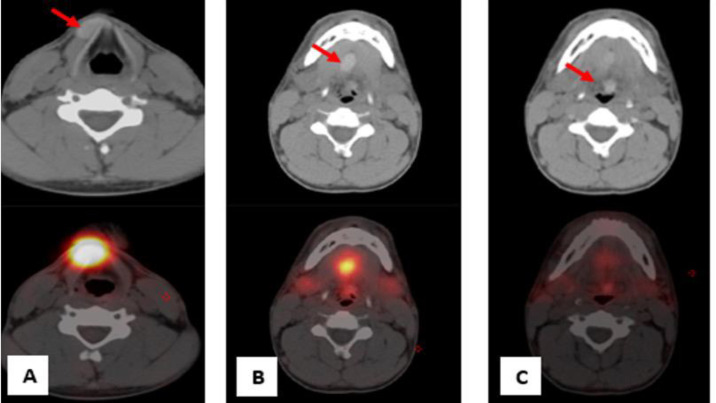
Axial non-contrast computed tomography and SPECT images showing hyperdense foci at (**A**) right infra-hyoid, (**B**) sublingual and (**C**) lingual regions (**red arrow**)

 The largest focus was observed on the right side just below the level of the hyoid bone (infra-hyoid), the second focus was situated between the geniohyoid and mylohyoid muscles (sublingual), and the third presented as a small focus with faint uptake at the base of the tongue (lingual) (Figure 2). Notably, no radiotracer uptake was evident in the normal thyroid bed. On the non-contrast SPECT images, the ectopic thyroid tissue appeared hyperdense (Figure 3).

## Discussion

 Ectopic thyroid gland is an uncommon embryological anomaly characterized by the presence of thyroid tissue in locations other than the typical anterior lower neck region (2). 

 It is a rare occurrence, affecting approximately 1 in 100,000 to 300,000 individuals (3). A comprehensive understanding of the embryological development of the thyroid is crucial in elucidating the etiology of ectopic thyroid locations. 

 The embryological development of the thyroid gland is a complex, well-orchestrated event that begins in the fetus around the end of the first month of gestation. It originates as a proliferation of endodermal cells on the foramen cecum at the base of the tongue, which is the site of the future thyroid gland. This thyroid primordium then descends ventrally in the neck through a transient, narrow canal known as the thyroglossal duct (4).The duct connects the developing thyroid gland to the original site of development at the base of the tongue.

 As the thyroid descends, it passes anterior to the hyoid bone and the laryngeal cartilages to reach its final pre-tracheal position, where it will bifurcate into two lobes connected by an isthmus. By approximately 7 weeks of gestation, the thyroglossal duct degenerates, and the thyroid begins to acquire its final shape and structure. The gland's descent is closely regulated by a series of genetic signals and morphogenic factors, such as the transcription factors TTF-1 (thyroid transcription factor 1), FOXE1 (forkhead box E1), and PAX8 (paired box gene 8), which play crucial roles in thyroid cell proliferation, differentiation, and migration (5).

 Disruptions in these genetic and embryonic processes can lead to ectopic thyroid tissue formation, which is when thyroid tissue does not reside in its typical location. The ectopic tissue results from thyroid cells failing to follow the normal migratory pathway or from remnants of the thyroglossal duct that did not properly degenerate and involute. These cells can then differentiate into fully functional thyroid tissue at any point along the migration path.

 The exact mechanism leading to ectopic thyroid tissue is not fully understood, but it is often attributed to genetic mutations or environmental factors that interfere with the migration or the vascularization of the thyroid gland. The presence of triple ectopic thyroid tissue, as in the case reported, suggests an even more complex disruption, as multiple sites of ectopic tissue imply multiple points of developmental arrest or aberrant differentiation.

 The locations mentioned, such as lingual, sublingual, or infra-hyoid, correspond to areas along the path of the thyroglossal duct. Lingual thyroid tissue is the most common form of ectopic thyroid, located at the base of the tongue, and results from a complete arrest of the thyroid primordium's descent. Sublingual ectopic thyroid tissue, found under the tongue, is less common and could indicate a later stage of descent before the arrest occurred. Infra-hyoid ectopic tissue suggests an even later disruption, closer to the final position of the thyroid (6). 

 Each ectopic focus may have varied functional capacity, often depending on the developmental stage at which the migratory process was interrupted. If the disruption occurs early in the embryonic development, the ectopic tissue might not have the full functional capacity typical of a fully developed thyroid gland. 

 Conversely, ectopic tissues that diverge later in the development may have more differentiated cells capable of normal thyroid function.

 In conclusion, the development of triple ectopic thyroid tissue formation is a rare embryological phenomenon that underscores the delicate balance of genetic and environmental factors in the migration and differentiation of thyroid cells. Understanding these intricate processes is essential for diagnosing and managing thyroid dysgenesis and its associated clinical implications.

 Thyroid scanning using Technetium-99m is the most sensitive diagnostic modality for detecting ectopic thyroid tissue; however, ultrasonography, CT scanning, and MRI may provide valuable insights into the extent and location of the ectopic thyroid gland (1). 

 Notably, ectopic thyroid tissue can mimic a thyroglossal duct cyst, as both can be located in the same anatomical position. Mistaking ectopic thyroid for a thyroglossal duct cyst and subsequently removing it can result in the ablation of all thyroid tissue, leading to physiological complications. Therefore, it is advisable to conduct a thyroid scan examination in all cases of thyroglossal duct cysts before removal to confirm the presence of a normal thyroid gland and prevent inadvertent total thyroid ablation, along with its potential medico-legal implications (7).

 Ectopic thyroid tissue shares the same characteristics as normal thyroid tissue and can be affected by similar conditions, including adenoma, hyperplasia, inflammation, goiterous enlargement, and even malignant trans-formation. Importantly, the risk of malignant transformation in ectopic thyroid tissue is not higher than that in normal thyroid Tissue (1).

 The clinical presentation of thyroid ectopia varies, with most cases being asymptomatic and incidentally discovered. Symptomatic cases depend on the location of the ectopic tissue and can include sensations of a foreign body, dysphagia, dysphonia, cough, or even hemoptysis in the case of lingual thyroid (8). 

 Asymptomatic cases are typically managed through observation with close follow-up. 

 Patients with hormone dysfunction may require hormone replacement therapy. Surgical removal should be considered when medical treatment fails, or there are obstructive symptoms, hemorrhage, or suspicion of malignancy. Notably, Man et al. reported a case of triple ectopic thyroid successfully treated with robotic surgery using a transoral and postauricular approach, avoiding the need for tracheostomy or other invasive open surgical procedures (3).

 While a few cases of dual ectopic thyroid have been reported, triple ectopic thyroid remains a rarer entity, with only a limited number of cases documented in the medical literature. An extensive review of existing literature reveals approximately 13 reported cases of triple ectopic thyroid. Table 1 summarizes these previously reported cases, considering factors such as age, gender, presenting symptoms, thyroid status, the presence or absence of a normal thyroid gland, ectopic thyroid location, and management. Interestingly, most of these cases were identified in females, with only two reported instances in male patients. Nearly all patients presented with a neck mass or swelling; however, our case stands out as a male patient who was entirely asymptomatic, with incidental subclinical hypothyroidism leading to the discovery of triple ectopic thyroid. 

 Furthermore, while there were slight variations in thyroid status among reported cases, the majority exhibited elevated TSH levels. Our patient's case is exceptionally rare, representing a male individual who remained asymptomatic and incidentally discovered to have triple ectopic thyroid.

**Table 1:** Comprehensive analysis of previously reported cases: age, gender, presenting symptoms, thyroid status, presence of normal thyroid gland, ectopic thyroid location, and management in incidental ectopic thyroid cases

**Authors (year)**	**Age (years)**	**Gender**	**Normal Thyroid gland**	**Thyroid status**	**Symptoms**	**Location**	**Management**
Konde et al. (2012) (2)	16	F	Absent	Normal (euthyroid)	Anterior midline neck swelling	Lingual, sublingual and infrahyoid	Follow up, then lost
Hua et al. (2019) (3)	56	F	Absent	Subclinical hypothyroidism	Firm midline neck mass	Lingual, submental region and anterior neck region	Surgical robotic excision (via Trans-Oral and Post-Auricular Approach)
Nilegaonkar et al. (2011) (7)	16	F	Absent	Hypothyroidism	Swelling in front of neck	Lingual, hyoid and suprahyoid regions	_
Mchonde et al. (2016) (9)	88	F	Present	_	Autopsy	Three inferior to the thyroid gland and extending to superior mediastinum	_
Ashok KG et al. (2021) (10)	20	F	Absent	Hypothyroidism	Swelling of anterior neck	Lingual, suprahyoid and infrahyoid	Thyroxine supplement and follow-up
Oikawa et al. (2013) (11)	10	F	Absent	Euthyroid at presentation, followed by hypothyroidism five months later	Neck swelling	Lingual, submandibular and pre-tracheal regions	Evothyroxine (75 µg/day)
Agrawal et al. (2019) (12)	11	F	Absent	Euthyroid	Midline neck swelling	Lingual, suprahyoid and infrahyoid	_
Rahalkar et al. (2014) (13)	42	F	Absent	Euthyroid	Nodule over the midline of the ck	Lingual, hyoid and infra-hyoid	_
Jain et al. (2010) (14)	20	F	Absent	Subclinical hypothyroidism	submandibular neck swelling	Lingual, suprahyoid and infrahyoid	_
Passah et al. (2018) (15)	5	F	Absent	Subclinical hypothyroidism	Midline neck swelling	Lingual, suprahyoid and pre-tracheal	_
Barai et al. (2004) (16)	6	M	Absent	Hypothyroidism	Midline neck swelling	Lingual, suprahyoid and cricoid cartilage	_
Nasiru et al. (2009) (17)	34	F	Present	Euthyroid	Lateral neck mass	Lateral neck region	Surgical excision
Pitts et al. (2022) (18)	46	M	Absent	Hypothyroidism	Globus sensation and left cervical LN	Lingual, sublingual and midline infrahyoid	Levothyroxine replacement therapy

## Conclusion

 In conclusion, triple ectopic thyroid tissue represents an exceptionally rare developmental anomaly, with only a few documented cases in the existing medical literature. This case underscores the remarkable rarity of such lesions, underscores the significance of ultrasound as an initial diagnostic tool, and emphasizes the pivotal role of Technetium-99m thyroid scanning as the gold standard imaging modality for pinpointing ectopic thyroid tissue in individuals with an absent orthotopic thyroid gland. A comprehensive understanding of this uncommon entity is essential for healthcare professionals, including physicians, surgeons, and radiologists, enabling accurate diagnosis and informed decisions regarding appropriate treatment strategies.
